# Association between metformin use and disease progression in obese people with knee osteoarthritis: data from the Osteoarthritis Initiative—a prospective cohort study

**DOI:** 10.1186/s13075-019-1915-x

**Published:** 2019-05-24

**Authors:** Yuanyuan Wang, Sultana Monira Hussain, Anita E. Wluka, Yuan Z. Lim, François Abram, Jean-Pierre Pelletier, Johanne Martel-Pelletier, Flavia M. Cicuttini

**Affiliations:** 1 Department of Epidemiology and Preventive Medicine, School of Public Health and Preventive Medicine, Monash University, 553 St Kilda Road, Melbourne, Victoria 3004 Australia; 2 Medical Imaging Research and Development, ArthroLab Inc., Montreal, Quebec, Canada; 3 Osteoarthritis Research Unit, University of Montreal Hospital Research Centre (CRCHUM), Montreal, Quebec, Canada

**Keywords:** Metformin, Osteoarthritis, Cartilage, Total knee replacement

## Abstract

**Objective:**

To examine whether metformin use was associated with knee cartilage volume loss over 4 years and risk of total knee replacement over 6 years in obese individuals with knee osteoarthritis.

**Methods:**

This study analysed the Osteoarthritis Initiative participants with radiographic knee osteoarthritis (Kellgren-Lawrence grade ≥ 2) who were obese (body mass index [BMI] ≥ 30 kg/m^2^). Participants were classified as metformin users if they self-reported regular metformin use at baseline, 1-year and 2-year follow-up (*n* = 56). Non-users of metformin were defined as participants who did not report the use of metformin at any visit from baseline to 4-year follow-up (*n* = 762). Medial and lateral cartilage volume (femoral condyle and tibial plateau) were assessed using magnetic resonance imaging at baseline and 4 years. Total knee replacement over 6 years was assessed. General linear model and binary logistic regression were used for statistical analyses.

**Results:**

The rate of medial cartilage volume loss was lower in metformin users compared with non-users (0.71% vs. 1.57% per annum), with a difference of − 0.86% per annum (95% CI − 1.58% to − 0.15%, *p* = 0.02), after adjustment for age, gender, BMI, pain score, Kellgren-Lawrence grade, self-reported diabetes, and weight change over 4 years. Metformin use was associated with a trend towards a significant reduction in risk of total knee replacement over 6 years (odds ratio 0.30, 95% CI 0.07–1.30, *p* = 0.11), after adjustment for age, gender, BMI, Kellgren-Lawrence grade, pain score, and self-reported diabetes.

**Conclusions:**

These data suggest that metformin use may have a beneficial effect on long-term knee joint outcomes in those with knee osteoarthritis and obesity. Randomised controlled trials are needed to confirm these findings and determine whether metformin would be a potential disease-modifying drug for knee osteoarthritis with the obese phenotype.

## Introduction

Osteoarthritis (OA) is one of the leading causes of global disability, resulting in over 12.8 million years lived with disability in 2015, an increase of 34.8% since 2005 [1]. Although OA is a multifactorial disease, management to date has taken a ‘one-size-fits-all’ approach that does not take into account the different pathological pathways and phenotypes of OA, resulting in a lack of disease-modifying agents and poor patient outcomes. Treatments that target the specific underlying pathological processes causing OA have the potential to affect disease progression. There is an urgent unmet need for a new approach that phenotypes individuals, enabling therapy with disease-modifying OA drugs targeted to those most likely to benefit.

One distinctive knee OA phenotype is the obese phenotype [2, 3], with disease mediated by mechanical, inflammatory and metabolic mechanisms [4, 5]. Over 50% of knee OA patients are obese [6]. Obesity and obesity-related inflammatory and metabolic factors (hyperglycaemia, dyslipidaemia and hypertension) are all risk factors for knee OA [4, 5]. Although some studies have suggested that type 2 diabetes might be a risk factor for knee OA, a recent systematic review has demonstrated that this is not independent of obesity [7]. Drugs targeting obesity and its associated inflammatory and metabolic abnormalities have the potential to slow the progression of knee OA. Metformin is a safe, well-tolerated oral biguanide widely used as first-line therapy for type 2 diabetes for over 50 years. In addition to its glucose-lowering effects, metformin modulates inflammatory and metabolic factors resulting in weight loss and reduced inflammation and plasma lipids [8]. Data from human studies suggest that metformin could reduce OA progression by modifying inflammatory and metabolic pathways. In randomised controlled trials of patients with symptomatic radiographic knee OA, combination of metformin and meloxicam resulted in greater reduction in serum levels of interleukin (IL)-1β, IL-8 and tumour necrosis factor (TNF)-α [9] and greater improvement in knee pain and function [10] compared with meloxicam alone over 12 weeks. In patients with OA and type 2 diabetes, a combination of cyclooxygenase (COX)-2 inhibitors and metformin reduced the rate of joint replacement over 10 years compared with COX-2 inhibitors alone [11]. Given the biological effects of metformin, knee OA patients with the obese phenotype represent the subgroup most likely to benefit from metformin which might be a potential disease-modifying agent in knee OA. Thus, the aim of our study was to determine whether metformin use was associated with a reduction in loss of knee cartilage volume over 4 years and risk of total knee replacement over 6 years in obese individuals with knee OA. It was hypothesised that metformin use would be associated with a reduced rate of cartilage volume loss and reduced risk of total knee replacement.

## Methods

### Participants

Osteoarthritis initiative (OAI) is a publicly available multicentre observational cohort study of knee OA (https://oai.nih.gov). The OAI comprises data of 4796 participants aged 45–79 years at baseline. OAI exclusion criteria included inflammatory arthritis, severe joint space narrowing in both knees, unilateral knee joint replacement and severe joint space narrowing in the contralateral knee, inability to undergo magnetic resonance imaging (MRI) or to provide a blood sample, required use of walking aids excepting a single straight cane ≤ 50% of the time, or unwilling to provide informed consent. Participants were recruited at four clinical sites (University of Maryland School of Medicine and Johns Hopkins University, Baltimore, Maryland; Ohio State University, Columbus, Ohio; University of Pittsburgh, Pittsburgh, Pennsylvania; Memorial Hospital of Rhode Island/Brown University, Pawtucket, Rhode Island), and the study was approved by the institutional review boards at each of the sites. All participants gave informed consent.

Bilateral standing posteroanterior fixed-flexion knee radiographs were assessed for baseline Kellgren- Lawrence (K-L) grading (0–4) (*n* = 4369). If only one knee had radiographic OA, this was selected for analyses. If both knees had radiographic OA, the most severe knee (i.e. highest K-L grade) was selected for analyses. When the severity was equal between sides, the most painful knee was selected for analyses. In the case of equal pain in both knees, the dominant knee was selected for analyses. The current study included participants with radiographic knee OA (K-L grade > 2) who were obese (body mass index (BMI) > 30 kg/m^2^), as K-L grade > 2 has been used as the definition of radiographic knee OA and has been included in typical entry criteria of knee OA clinical trials.

### Metformin use and diabetes

OAI participants were required to bring their medications when attending the baseline and yearly follow-up study visits, with medication name, duration and frequency of use (as needed or regular) recorded. Participants were classified as metformin users if they self-reported regular metformin use at baseline, 1-year and 2-year follow-up. Non-users of metformin were defined as participants who did not report the use of metformin at any visit from baseline to 4-year follow-up. Data on self-reported diabetes was collected using a questionnaire at baseline, 2-year and 4-year follow-up.

### Knee pain

At baseline and yearly follow-up, knee pain was assessed using the Western Ontario and McMaster Universities Osteoarthritis Index (WOMAC) pain subscale [12] Likert scale version. It consists of five items, with the score ranging 0–20.

### MRI and cartilage volume measurement

MRI was performed for the target knee using a 3 T scanner (Magnetom Trio, Siemens, Erlangen, Germany) and the exam consisted of a sagittal double-echo in steady-state (DESS) sequence. Knee cartilage volume was measured for medial and lateral tibiofemoral compartments (i.e. femoral condyle and tibial plateau) using the automatic human knee cartilage segmentation as previously described and validated, delineated as previously described and implemented in the automated segmentation [13]. The correlation between the automatic and the semi-automatic segmentations was excellent with Pearson correlations of *r* = 0.96 (*p* < 0.0001) for the global knee, *r* = 0.95 (*p* < 0.0001) for the femur, and *r* = 0.83 (*p* < 0.0001) for the tibia. The test-retest revealed an excellent measurement error of 0.3 ± 1.6% for the global knee (0.14 ± 1.7% for the femur and 1.12 ± 3.8% for the tibial plateau), corresponding to 30.3 ± 126.2 mm^3^ [13]. The annual rate of cartilage volume loss over 4 years was obtained by (4-year follow-up volume - baseline volume)/baseline volume/4, expressed as a percentage.

### Total knee replacement

At each available follow-up, participants indicated whether they had received total knee replacement surgery. It was defined as any knee with patient-reported total knee replacement which was confirmed on subsequent radiograph between baseline and 6-year follow-up visit. Missing data for a knee replacement was treated conservatively by assuming that the participant had not undergone knee replacement surgery.

### Statistical analyses

Demographic, clinical, radiological and MRI data were systematically entered into a computerised database. Descriptive statistics of participant characteristics were tabulated and compared between metformin users and non-users using independent samples *t* test or chi-square test, as appropriate. The rates of cartilage volume loss over 4 years were compared between metformin users and non-users using *F*-test (general linear model) with estimated marginal means (standard error, SE), adjusted for gender, baseline age, BMI, K-L grade, WOMAC pain score, diabetes, and weight change over 4 years. Binary logistic regression was used to examine the association between metformin use and risk of total knee replacement over 6 years, adjusted for gender, baseline age, BMI, K-L grade, WOMAC pain score, and diabetes. All tests were two-sided and a *p* value < 0.05 was considered statistically significant. Statistical analyses were performed using Stata 13.0 (StataCorp LP., College Station, TX, USA).

## Results

Participant characteristics are shown in Table 1. Among 818 participants included in the current study, 56 (6.8%) were metformin users. Metformin users had higher BMI (34.9 kg/m^2^ vs. 33.9 kg/m^2^), experienced greater knee pain (5.8 vs. 4.3) and more likely to have self-reported diabetes (98.2% vs. 8.9%) compared with non-users. There was no significant difference in age, gender, or K-L grade between the two groups. There were 540 (66.0%) participants having knee cartilage volume measured at 4-year follow-up. There were no significant differences in terms of baseline age, gender, BMI, diabetes, metformin use, or weight change over 4 years between participants with and without knee cartilage volume assessed at 4-year follow-up. Those without knee cartilage volume assessed at 4-year follow- up had greater knee pain and were more likely to have severe knee OA (K-L grade 4) at baseline.

**Table 1:** Characteristics of study participants

	Metformin users *N* = 56	Non-users *N* = 762	p*
Baseline data			
Age, years	62.3 (7.7)	61.7 (8.5)	0.64
Female, *n* (%)	41 (73.2)	492 (64.6)	0.19
Body mass index, kg/m^2^	34.9 (3.8)	33.9 (3.1)	0.02
WOMAC pain score	5.8 (4.4)	4.3 (4.1)	0.01
Kellgren-Lawrence grade, *n* (%)			0.93
2	28 (50.0)	386 (50.7)	
3	21 (37.5)	293 (38.4)	
4	7 (12.5)	83 (10.9)	
Longitudinal data over 4–6 years			
Self-reported diabetes over 4 years, *n* (%)	55 (98.2)	68 (8.9)	< 0.001
Change in weight over 4 years, kg (*n* = 674)	− 1.4 (8.4)	− 0.6 (6.6)	0.45
Annual % loss in cartilage volume over 4 years (*n* = 540)			
Medial compartment	1.15 (1.29)	1.53 (1.63)	0.19
Lateral compartment	1.31 (1.12)	1.20 (1.40)	0.65
Total knee replacement over 6 years, *n* (%)	3 (5.4)	88 (11.6)	0.16

Data presented as mean (standard deviation) or no (%)

*For the difference between metformin users and non-users using independent samples *t* test or chi-square test

The annual rate of medial cartilage volume loss was lower in metformin users than in non-users after adjustment for age, gender, BMI, K-L grade, WOMAC pain score, and diabetes [0.83% (SE 0.34) vs. 1.55% (SE 0.07), *p* = 0.04] (Table 2). To explore whether the difference in the rate of cartilage volume loss was explained by the greater weight loss observed in the metformin users, additional adjustment for weight change over 4 years was performed. The rate of medial cartilage volume loss was still lower in metformin users compared with non-users [0.71% (SE 0.35) vs. 1.57% (SE 0.07) per annum], with a difference of − 0.86% per annum (95% confidence interval (CI) − 1.58 to − 0.15%, *p* = 0.02). The rate of lateral cartilage volume loss was not significantly different between the two groups (Table 2).

**Table 2:** Association between metformin use and annual percentage loss in knee cartilage volume over 4 years

	Metformin users (mean, SE)^1^	Non-users (mean, SE)^1^	Difference (mean, 95% CI)^1^	*P* ^1^	Metformin users (mean, SE)^2^	Non-users (mean, SE)^2^	Difference (mean, 95% CI)^2^	*P* ^2^
Medial cartilage volume loss (%)	0.83 (0.34)	1.55 (0.07)	− 0.72 (− 1.42, − 0.02)	0.04	0.71 (0.35)	1.57 (0.07)	− 0.86 (− 1.58, − 0.15)	0.02
Lateral cartilage volume loss (%)	0.98 (0.29)	1.22 (0.06)	− 0.24 (− 0.85, 0.36)	0.43	1.03 (0.28)	1.24 (0.06)	− 0.20 (− 0.79, 0.38)	0.49

*SE* standard error, *CI* confidence interval

^1^Adjusted for age, gender, body mass index, Kellgren-Lawrence grade, WOMAC pain score, and diabetes

^2^Adjusted for age, gender, body mass index, Kellgren-Lawrence grade, WOMAC pain score, diabetes, and weight change over 4 years

Three (5.4%) metformin users and 88 (11.6%) non-users underwent total knee replacement over 6 years. Metformin use was associated with a non- significant reduction in the risk of total knee replacement over 6 years in unadjusted analysis (odds ratio 0.43, 95% CI 0.13–1.42, *p* = 0.17), and after adjustment for age, gender, BMI, K-L grade, WOMAC pain score, and diabetes (odds ratio 0.30, 95% CI 0.07–1.30, *p* = 0.11).

In this community-based cohort, the mean WOMAC pain scores were not very high across the study period (out of 20): 4.4 (standard deviation, SD, 4.1) at baseline; 3.8 (SD 3.9) at 1-year follow-up; 3.7 (SD 3.8) at 2-year follow-up; 3.8 (SD 4.0) at 3-year follow-up; and 3.7 (SD 3.9) at 4-year follow-up. There was a minimal change in WOMAC pain score over 4 years in metformin users and non-users [− 0.9 (SD 4.2) vs. − 0.6 (SD 3.8), *p* = 0.54], with no clinical or statistically significant difference between the two groups. The result was similar after adjustment for age, gender, BMI, K-L grade, WOMAC pain score, diabetes, and weight change over 4 years [− 0.9 (SE 0.6) vs. − 0.5 (SE 0.1), *p* = 0.58].

Additional analyses were performed in obese participants with or at risk for knee OA. The annual rate of medial cartilage volume loss was lower in metformin users compared with non-users [0.67% (SE 0.26) vs. 1.23% (SE 0.05)], with a difference of − 0.56% (95% CI − 1.08 to − 0.04%, *p* = 0.03), after adjustment for age, gender, BMI, K-L grade, WOMAC pain score, diabetes, and weight change over 4 years.

## Discussion

In those with knee OA and obesity, metformin use was associated with a reduced rate of medial knee cartilage volume loss over 4 years and a trend towards a significant reduction in risk of total knee replacement over 6 years, after adjusting for potential confounders including weight, knee pain, diabetes, and severity of knee OA.

Metformin is a well-tolerated drug with pleiotropic effects and a long history of safe use in diverse patient groups, including pre- and non-diabetic populations [8]. However, there have been limited data examining the effect of metformin use on joint outcomes. In a randomised, double-blinded clinical study of patients with symptomatic and radiological evidence of knee OA, the combination of metformin and meloxicam resulted in greater improvement in all the components of Knee Injury and Osteoarthritis Outcome Score compared with meloxicam alone [10]. Similarly, a nationwide, retrospective, matched-cohort study of patients with OA and type 2 diabetes in Taiwan found that metformin combined with COX-2 inhibitors reduced the risk of joint replacement surgery by 25% over 10 years compared with COX-2 inhibitors alone [11]. In contrast, another study reported no association between metformin use and general practitioner electronic diagnosis of OA [14]. As this diagnosis of OA may not be an accurate and validated method for identifying OA, it provides a potential explanation for the non-significant results from the study. Our study has extended the previous studies by investigating the association between metformin use and knee joint outcomes in a subgroup of people with the specific obese knee OA phenotype who are most likely to respond. We found consistent results for a relationship between metformin use and reduced knee OA progression, evidenced by a decreased rate of medial cartilage volume loss and decreased risk of total knee replacement, although the association for the latter was not statistically significant. The medial tibiofemoral compartment is most commonly affected by OA, and cartilage volume in this compartment is sensitive to change, correlates with worsening symptoms and radiographic progression, and predicts knee replacement [15]. Our study did not show an association between metformin use and change in WOMAC pain over 4 years. This might be due to the mild knee pain in this cohort which remained relatively stable over the study course in both metformin users and non-users, and thus, our study did not have the power to show a significant difference in change in WOMAC pain between the two groups; the higher rate of total knee replacement in non-users than metformin users may have underestimated the difference in knee pain between the two groups.

We found a beneficial effect of metformin on preserving knee cartilage, independent of weight loss, suggesting this is not the sole mechanism. The mechanisms may be via metformin’s effect on modulating inflammatory and metabolic pathways which in turn reduces inflammation and lowers plasma glucose and lipids [8]. In murine arthritis models, metformin attenuated arthritis scores and bone destruction and reduced serum levels of pro-inflammatory cytokines and immunoglobulin [16, 17]. Elevated IL-6 and TNF-α levels [18] and greater fasting glucose levels [19] were associated with an increased rate of tibial cartilage volume loss. Elevated cholesterol and triglyceride levels were associated with an increased incidence of bone marrow lesions, an early structural change in knee OA [20]. By targeting these multiple metabolic and inflammatory pathways, metformin would have an effect on reducing the rate of knee cartilage volume loss. Data from our study, together with data from previous human [9–11] and animal [16, 17] studies, provide proof of concept that targeting obesity and obesity-related inflammatory, and metabolic pathways may have a disease-modifying effect in knee OA and that metformin might be a potential disease-modifying OA drug by affecting multiple pathological pathways in people with knee OA who are obese. Randomised controlled trials are needed to clarify this.

This study has potential limitations. Metformin use was defined based on regular use of metformin medication at baseline, 1-year and 2-year follow-up. Metformin use was self-reported with information on duration and frequency of use available in the OAI. However, data related to dosage and compliance were not reported in the OAI. This may have resulted in misclassification of metformin use. However, this is most likely to have resulted in non-differential misclassification and thus underestimated the magnitude of the observed associations. There are issues of confounding by indication and selection bias related to prescription medication use in observational studies. In the current study, participants using metformin were similar to non-users in terms of age, gender and severity of knee OA, but had higher BMI and greater knee pain, and were more likely to have diabetes. So any bias related to metformin use would have tended to result in worse outcome in the metformin users rather than the beneficial effect observed. We have adjusted for BMI, WOMAC pain score and self-reported diabetes in the statistical analyses. For the incidence of total knee replacement, we have assumed that those lost to follow-up did not have a total knee replacement. These would have resulted in non-differential misclassification of total knee replacement, leading to underestimation of the magnitude of observed associations. As the number of metformin users with a total knee replacement was small, the study had limited power to detect a significant association between metformin use and risk of total knee replacement; therefore, the results should be considered suggestive. The present study also has strengths. The OAI offered a unique opportunity to study the disease profile of a large number of participants with or at risk for knee OA and explore the longitudinal association between medication use and long-term joint outcomes. The use of fully automated technologies to assess knee cartilage volume change over time [13] greatly improved the capacity and more importantly the reliability of the analysis.

## Conclusions

Our data suggest that metformin use may have a beneficial effect on long-term knee joint outcomes in obese knee OA patients, evidenced by a reduced rate of medial cartilage volume loss and a trend towards a significant reduction in the risk of total knee replacement. Randomised controlled trials are needed to determine whether metformin would be a disease-modifying drug for knee OA with the obese phenotype.

## Abbreviations

BMI: Body mass index
CI: Confidence interval
COX: Cyclooxygenase
DESS: Double-echo in steady-state
IL: Interleukin
K-L: Kellgren-Lawrence
MRI: Magnetic resonance imaging
OA: Osteoarthritis
OAI: Osteoarthritis Initiative
SE: Standard error
TNF: Tumour necrosis factor
WOMAC: Western Ontario and McMaster Universities Osteoarthritis Index

## References

[1] GBD 2015 Disease and Injury Incidence and Prevalence Collaborators. Global, regional, and national incidence, prevalence, and years lived with disability for 310 diseases and injuries, 1990–2015: a systematic analysis for the Global Burden of Disease Study 2015. Lancet 2016, 388:1545–1602. 10.1016/S0140-6736(16)31678-6 PMC5055577 27733282

[2] van der Esch M, Knoop J, van der Leeden M, Roorda LD, Lems WF, Knol DL, Dekker J (2015). Clinical phenotypes in patients with knee osteoarthritis: a study in the Amsterdam osteoarthritis cohort. Osteoarthr Cartil.

[3] Knoop J, van der Leeden M, Thorstensson CA, Roorda LD, Lems WF, Knol DL, Steultjens MP, Dekker J (2011). Identification of phenotypes with different clinical outcomes in knee osteoarthritis: data from the osteoarthritis initiative. Arthritis Care Res (Hoboken).

[4] Thijssen E, van Caam A, van der Kraan PM (2015). Obesity and osteoarthritis, more than just wear and tear: pivotal roles for inflamed adipose tissue and dyslipidaemia in obesity-induced osteoarthritis. Rheumatology (Oxford).

[5] Berenbaum F, Eymard F, Houard X (2013). Osteoarthritis, inflammation and obesity. Curr Opin Rheumatol.

[6] Puenpatom RA, Victor TW (2009). Increased prevalence of metabolic syndrome in individuals with osteoarthritis: an analysis of NHANES III data. Postgrad Med.

[7] Dawson LP, Fairley JL, Papandony MC, Hussain SM, Cicuttini FM, Wluka AE (2018). Is abnormal glucose tolerance or diabetes a risk factor for knee, hip, or hand osteoarthritis? A systematic review. Semin Arthritis Rheum.

[8] Saisho Y (2015). Metformin and inflammation: its potential beyond glucose-lowering effect. Endocr Metab Immune Disord Drug Targets.

[9] Mohammed MM, Al-Shamma K, Jasim NA (2014). Evaluation of the anti-inflammatory effect of metformin as adjuvant therapy to NSAID (meloxicam) in patients with knee osteoarthritis. Int J Sci Nature.

[10] Mohammed MM, Al-Shamma KJ, Jassim NA (2014). Evaluation of the clinical use of metformin or pioglitazone in combination with meloxicam in patients with knee osteoarthritis; using knee injury and osteoarthritis outcome score. Iraqi J Pharm Sci.

[11] Lu CH, Chung CH, Lee CH, Hsieh CH, Hung YJ, Lin FH, Tsao CH, Hsieh PS, Chien WC (2018). Combination COX-2 inhibitor and metformin attenuate rate of joint replacement in osteoarthritis with diabetes: a nationwide, retrospective, matched-cohort study in Taiwan. PLoS One.

[12] Bellamy N, Buchanan WW, Goldsmith CH, Campbell J, Stitt LW (1988). Validation study of WOMAC: a health status instrument for measuring clinically important patient relevant outcomes to antirheumatic drug therapy in patients with osteoarthritis of the hip or knee. J Rheumatol.

[13] Dodin P, Pelletier JP, Martel-Pelletier J, Abram F (2010). Automatic human knee cartilage segmentation from 3D magnetic resonance images. IEEE Trans Biomed Eng.

[14] Barnett LA, Jordan KP, Edwards JJ, van der Windt DA (2017). Does metformin protect against osteoarthritis? An electronic health record cohort study. Prim Health Care Res Dev.

[15] Pelletier JP, Cooper C, Peterfy C, Reginster JY, Brandi ML, Bruyere O, Chapurlat R, Cicuttini F, Conaghan PG, Doherty M (2013). What is the predictive value of MRI for the occurrence of knee replacement surgery in knee osteoarthritis?. Ann Rheum Dis.

[16] Kang KY, Kim YK, Yi H, Kim J, Jung HR, Kim IJ, Cho JH, Park SH, Kim HY, Ju JH (2013). Metformin downregulates Th17 cells differentiation and attenuates murine autoimmune arthritis. Int Immunopharmacol.

[17] Son HJ, Lee J, Lee SY, Kim EK, Park MJ, Kim KW, Park SH, Cho ML (2014). Metformin attenuates experimental autoimmune arthritis through reciprocal regulation of Th17/Treg balance and osteoclastogenesis. Mediat Inflamm.

[18] Stannus O, Jones G, Cicuttini F, Parameswaran V, Quinn S, Burgess J, Ding C (2010). Circulating levels of IL-6 and TNF-alpha are associated with knee radiographic osteoarthritis and knee cartilage loss in older adults. Osteoarthr Cartil.

[19] Davies-Tuck ML, Wang Y, Wluka AE, Berry PA, Giles GG, English DR, Cicuttini FM (2012). Increased fasting serum glucose concentration is associated with adverse knee structural changes in adults with no knee symptoms and diabetes. Maturitas.

[20] Davies-Tuck ML, Hanna F, Davis SR, Bell RJ, Davison SL, Wluka AE, Adams J, Cicuttini FM (2009). Total cholesterol and triglycerides are associated with the development of new bone marrow lesions in asymptomatic middle-aged women - a prospective cohort study. Arthritis Res Ther.

